# BananaSet: A dataset of banana varieties in Bangladesh

**DOI:** 10.1016/j.dib.2024.110513

**Published:** 2024-05-11

**Authors:** Md Ripon Sheikh, Md. Anwar Hossain, Moazzem Hossain, Md. Masudul Islam, Galib Muhammad Shahriar Himel

**Affiliations:** a Department of Computer Science and Engineering, Bangladesh University of Business and Technology (BUBT), Dhaka, Bangladesh; b Department of Computer Science, American International University-Bangladesh, Dhaka, Bangladesh

**Keywords:** Banana Types, Image Dataset, Classification, Image Processing, Fruit Classification, Deep Learning, Agriculture, Taxonomy

## Abstract

This article introduces a primary dataset sourced from diverse marketplaces in Bangladesh, encompassing six distinct banana varieties predominantly consumed locally. The dataset comprises the following banana types: *Shagor, Shabri, Champa, Anaji, Deshi, and Bichi*. High-resolution images of bananas from each category were acquired using a smartphone camera. A total of 1166 images were captured but did not maintain a uniform distribution. Only the augmented data has 1000 images per category which is a total of 6000 images. The proposed dataset exhibits substantial potential for impact and utility, offering a range of attributes, including but not limited to the representation of six diverse banana varieties, each possessing unique flavors and holding promise for various applications within the agriculture and food manufacturing industries.

Specifications Table

**Table:** 

Subject	Computer Science
Specific subject area	Computer Vision, Pattern Recognition, machine learning, deep learning
Data format	Raw
Type of data	JPG Image
Description of data collection	This dataset presents a collection of high-resolution images showcasing six popular banana varieties sourced from two distinct regions in Bangladesh. These bananas were meticulously sampled from both rural orchards and local markets, providing a rich and diverse representation. The dataset serves as a visual compendium, offering a comprehensive view of the various aspects of these banana types, aiding in their precise classification. It encompasses six distinctive classes, specifically, Shagor, Shabri, Champa, Anaji, Deshi, and Bichi, with a combined total of 1166 raw images and 6000 augmented images. Each of these banana varieties was diligently captured during the period spanning from August 01 to August 15, 2023. It is worth emphasizing the unique nature of this dataset, as it stands as an entirely novel resource, previously unused by any prior research endeavors.
How the data were acquired	Smartphone Camera
Data source location	**Location:** Faridpur & Dhaka **Zone:** Binokdia & Mirpur-1 **Country:** Bangladesh
Data accessibility	**Repository name:** Mendeley Data **Data identification number:**10.17632/35gb4v72dr.4 **Direct URL to data**: https://data.mendeley.com/datasets/35gb4v72dr

## 1 Value of the Data

• This fresh dataset containing images of Banana fruits can serve as the foundation for creating an automated classification system within the industry, addressing the classification challenge effectively.
• It can serve as a critical resource for agricultural research, enabling the identification and classification of distinct banana types, and aiding in crop management.
• This dataset has relevance in machine learning, fostering the development of image recognition models and enhancing automated fruit sorting systems.
• Farmers and agricultural experts can use it to enhance the precision of banana variety identification and crop management, ultimately improving agricultural yields.
• The proposed dataset has the potential to be of great utility to the computer science community, especially within the domains of computer vision, machine learning, and deep learning. It can be instrumental in the creation of robust banana classification models capable of accurately distinguishing between different banana types.
• Such models offer the farming community a valuable resource to optimize their pre-planting processes, saving time and resources involved in cultivating specific banana varieties while mitigating the risks associated with planting the wrong types.

## 2 Background

Musaceae or Bananas, known as the “fruit of the wise,” are cherished for their tropical sweetness and nutritional value. Rich in potassium and fiber, they provide energy and flavor. Given that bananas can thrive in a variety of nations and are available throughout the year, they have emerged as the fourth most significant climacteric fruit commodity [1]. This makes them suitable for both domestic consumption and exportation [2]. Banana cultivation involves planting shoots, and their adaptability to warm climates sustains countless livelihoods. Beyond their deliciousness, bananas feature various culinary creations and offer substantial nutrition, affirming their significance in both agriculture and culinary traditions worldwide.

## 3 Data Description

In our paper, we proposed a dataset that comes with two variations: Raw images folder and Augmented images Folder. Each one is organized into six distinct sub-folders, each corresponding to a specific banana variety. Within the original image folder, there are 1166 images in JPG format. These images are uniformly set and at a resolution of 4608 × 3456 pixels. The high resolution of the images initially resulted in a considerable file size of 4.08 GB. After applying compression through a zip program, the dataset size was further optimized to 3.55 GB. Subsequently, data augmentation techniques were applied, as deep learning models for machine vision necessitate a substantial volume of images. Augmentation was executed by implementing transformations such as scaling, shifting, shearing, zooming, and random rotation. The specific augmentation parameters employed included rotations at angles of 1° to 40°, with width, height shift, zoom range, and shear ranges set to 0.2. As a result, from the raw images in each class, we generated an additional 1000 augmented images, culminating in a dataset comprising a total of 6000 augmented images (1000 per class). Within the augmented image folder, there are 6000 images in JPG format. These images are uniformly set and at a resolution of 4608 × 3456 pixels where the main size is 4.73 GB but after compression, this was optimized to 4.46 GB after compression.

The process of dataset generation is visually depicted in Fig. 2, while Fig. 3 provides a visual representation of augmented images derived from an original sample image. The banana dataset is conveniently accessible through the Mendeley repository [3], where it is stored in two distinct zip files, namely '*Augmented_Images.zip*' and '*Original_Images.zip*'.

**Fig. 1:**
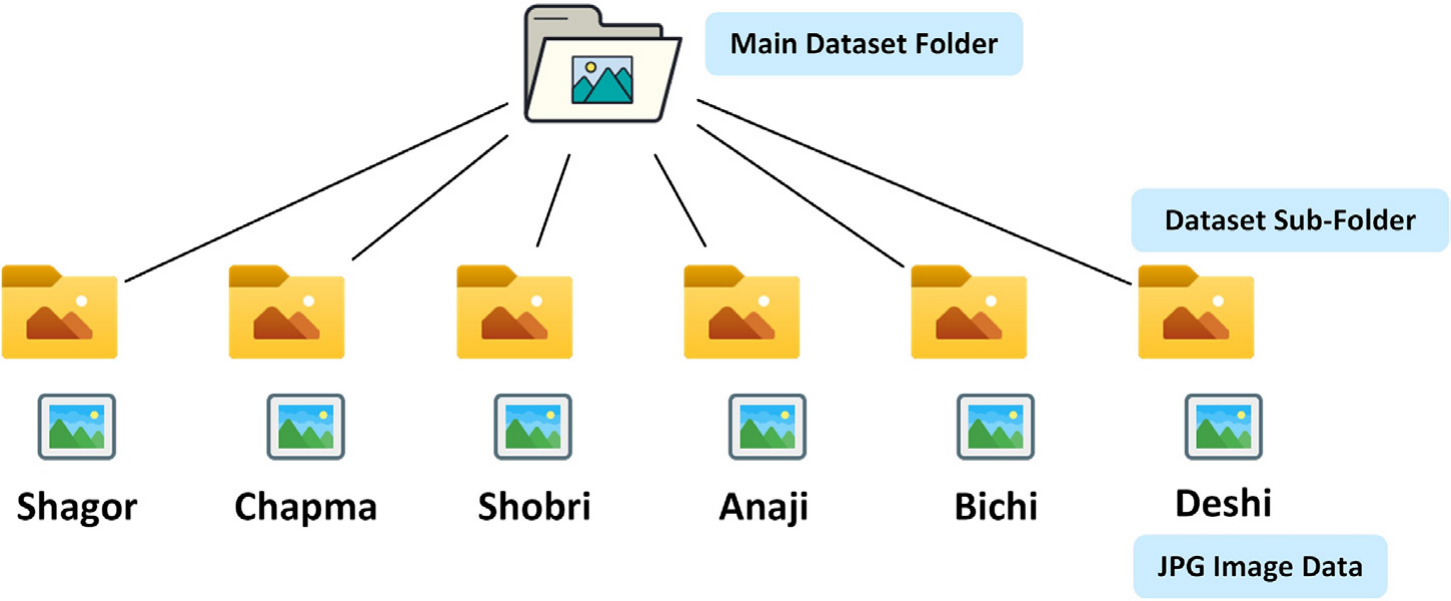
Dataset folder structure.

Both zip files contain six folders representing a unique banana variety, namely *Shagor, Shabri, Champa, Anaji, Deshi, and Bichi*. Some of these banana types have official names such as, *Shagor* is *BARI-1, Anaji* is *BARI-2*, and *Deshi* is *BARI-4*. [4]. Fig. 1 is a visual representation of our raw and augmented dataset structure.

**Fig. 2:**
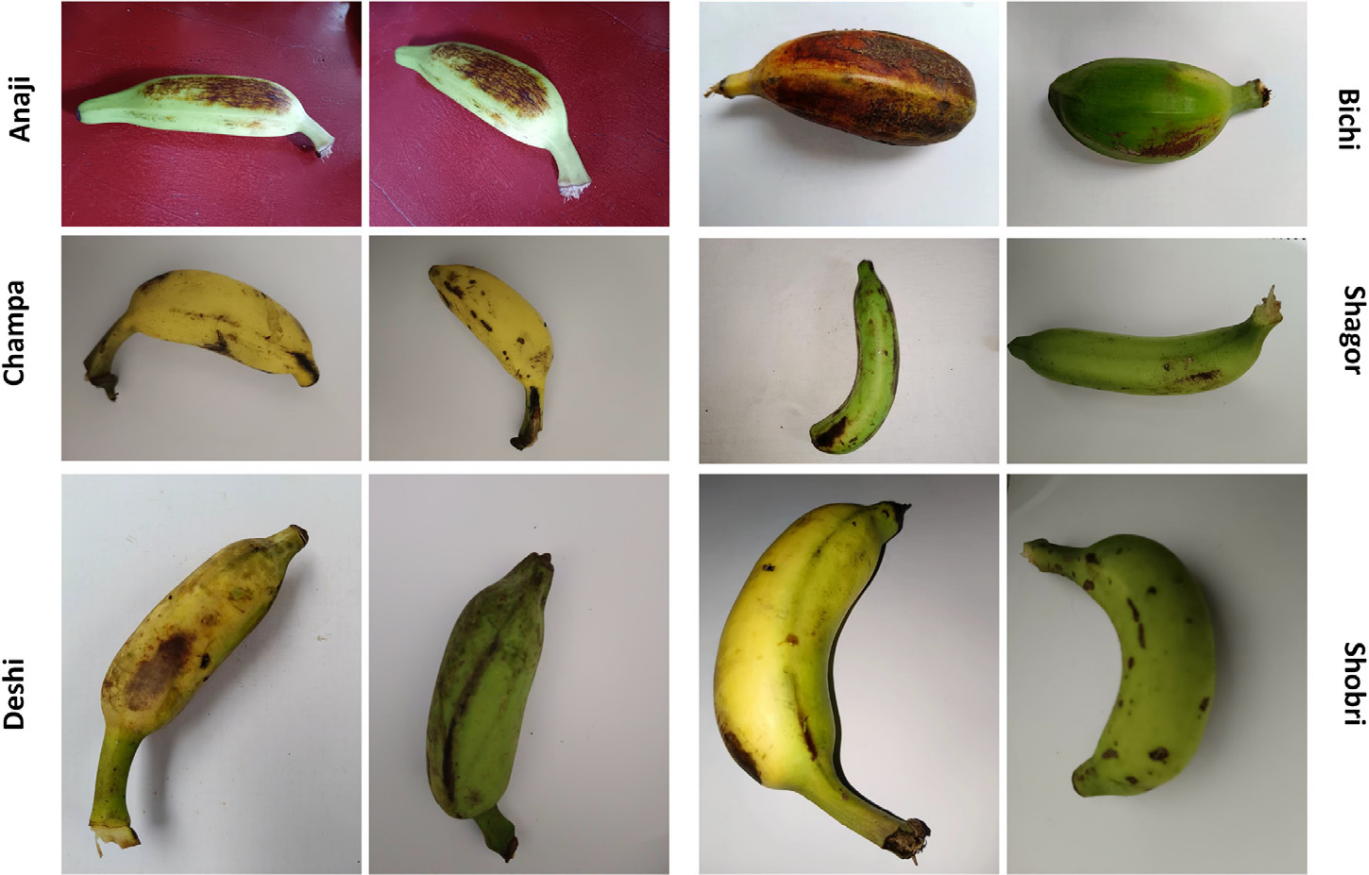
Examples of each of the Banana class.

**Fig. 3:**
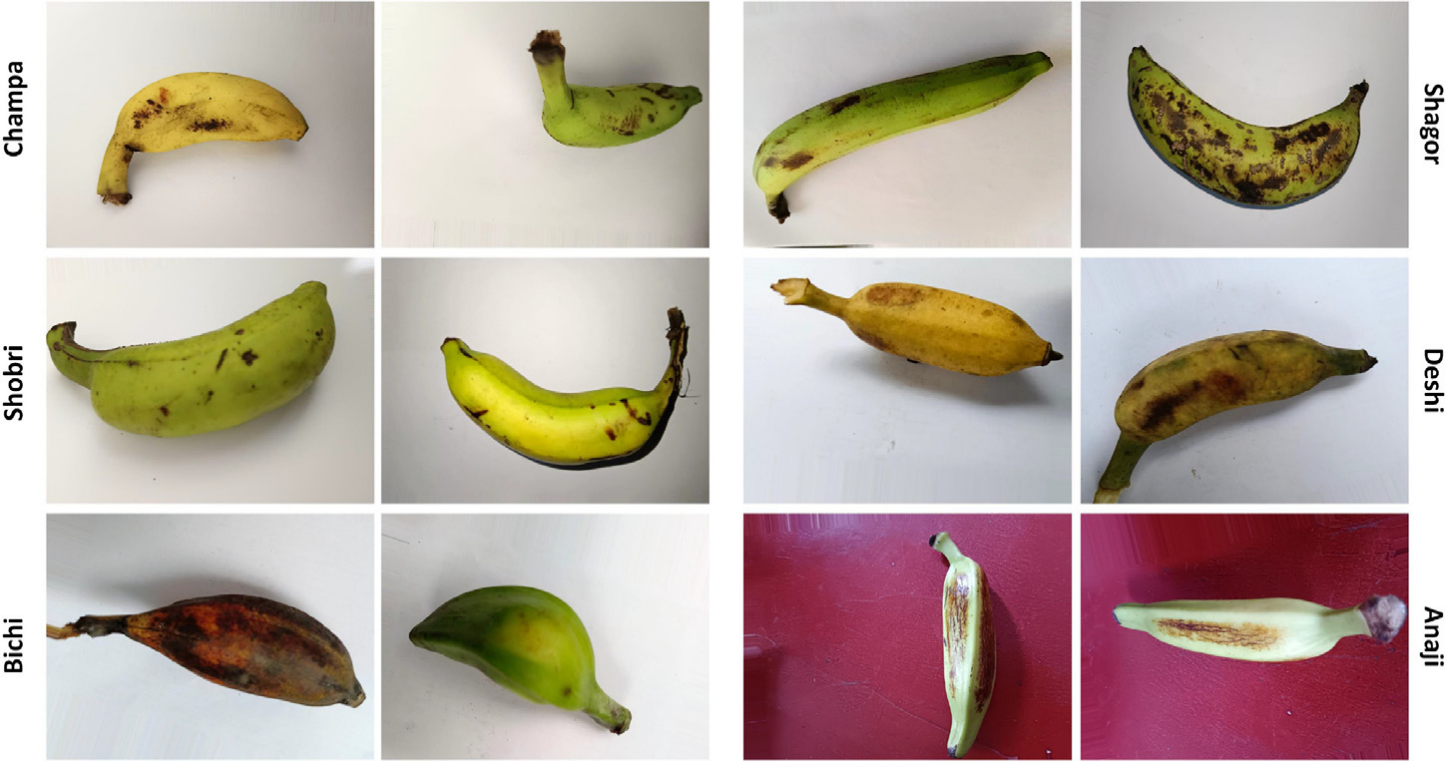
Examples of augmented dataset.

This dataset has the potential to act as a state-of-the-art guide for the development of machine vision algorithms designed to classify various banana types within the agricultural domain.

## 4 Experimental Design, Materials and Methods

**Fig. 4:**
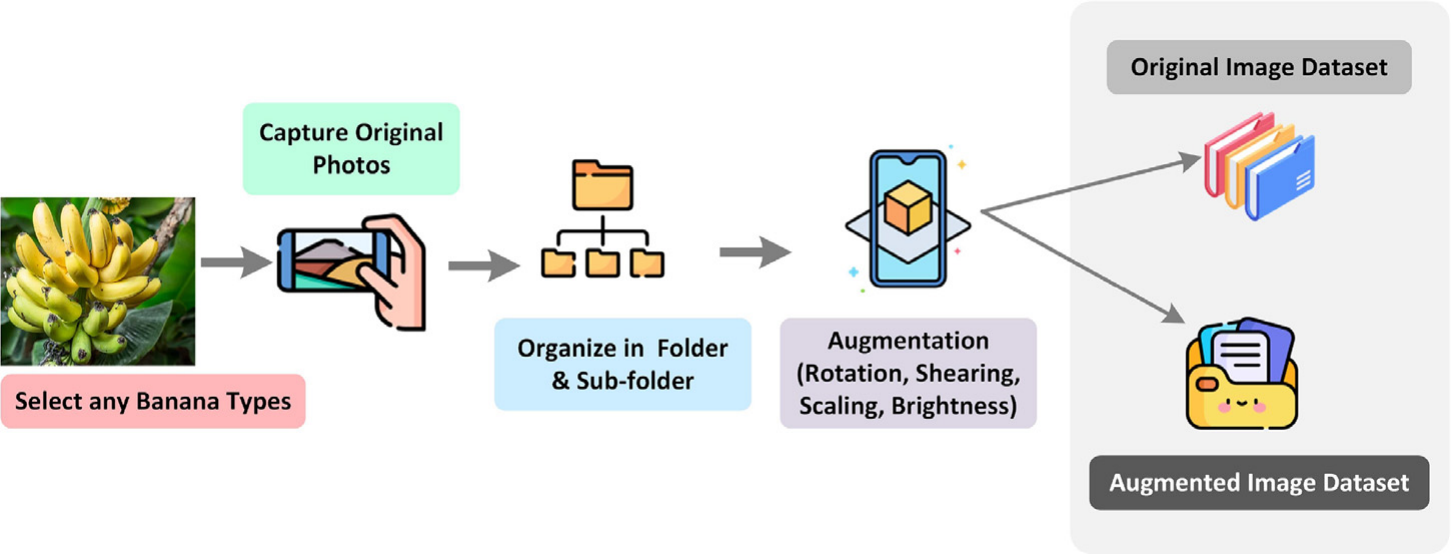
Workflow for the banana varieties dataset generation.

The process of acquiring images for each type of banana adhered to a specific workflow, which is visualized in Fig. 4. In this method, the selection of individual bananas from each type of banana population followed a uniform distribution, ensuring that each banana type had an equal probability of being selected. This approach was instrumental in maintaining a diverse representation of data. To achieve this diversity, a random selection process was employed. Throughout the data collection phase, several challenges were encountered. Given that the bananas were sourced from naturally growing trees and local markets, a notable portion exhibited various stages of decay, partial consumption by bats or worms, or damage, posing potential obstacles to the identification process. Consequently, bananas displaying deformities were intentionally excluded from the dataset. Each banana chosen for photography was selected randomly from within a cluster of bananas. After the images of each banana type were captured, they were subsequently transferred from the smartphone's memory to an external hard drive. These images were then organized within folders, each folder bearing the name of the corresponding banana type. To label the dataset correctly local experts were involved along with a domain expert. After confirmation, the local names were used for labeling the data as local names are more popular than the official names. The process of acquiring images for the subsequent banana type commenced once the images of the previously captured banana type were removed from the smartphone's memory. This systematic approach continued until images for all categories of bananas were obtained, ranging from raw to ripe, ensuring a comprehensive representation of the dataset. Table 1 shows the statistical view of our dataset.

**Table 1:** Statistics of the banana varieties dataset.

Category (Local name)	Official name	Number of original images	Number of augmented images
Shagor	*BARI-1*	239	1000
Shobri	*Shobri*	163	1000
Champa	*Champa*	136	1000
Anaji	*BARI-2*	209	1000
Deshi	*BARI-4*	237	1000
Bichi	*Bichi*	182	1000
Total		**1166**	**6000**

### 4.1 Camera Specification

**Table 2:** Represents the VIVO V25 5G smartphone camera.

Triple	64 MP, f/1.8, (wide), 0.7 µm, PDAF, OIS 8 MP, f/2.2, 16 mm, 120° (ultrawide), 1/4″, 1.12 µm 2 MP, f/2.4, (macro)
Features	LED flash, HDR, panorama

### 4.2 Model validation

**Fig. 5:**
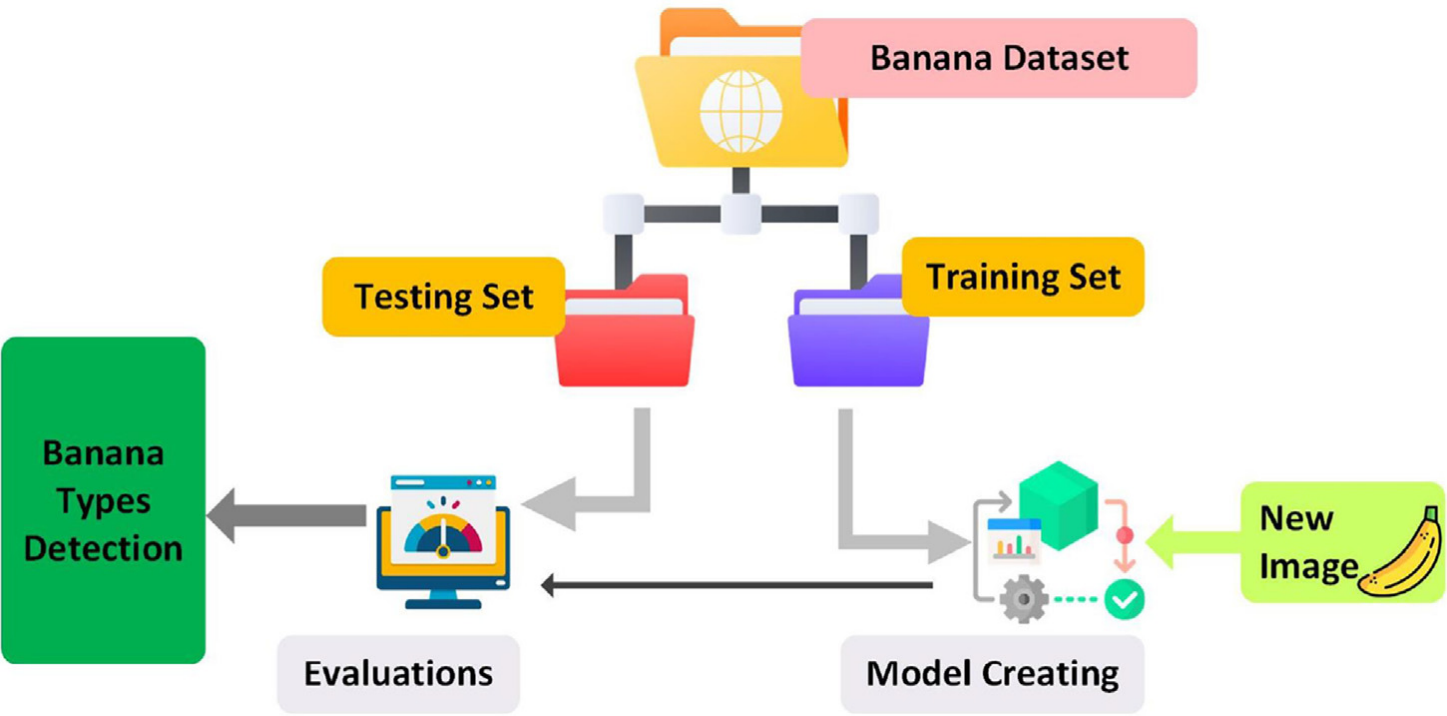
Generic method for banana varieties identification.

**Fig. 6:**
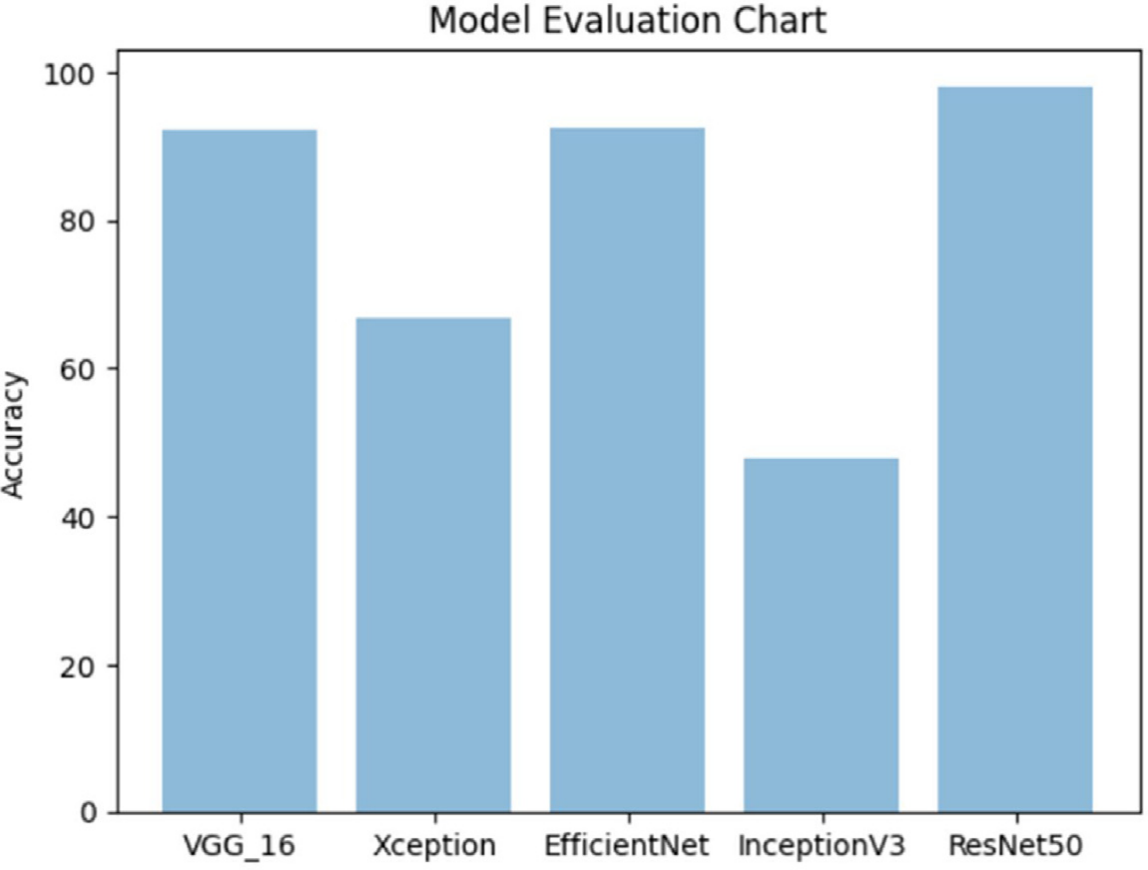
Accuracy comparison.

We present a traditional deep learning model for the efficient training of the dataset, resulting in cutting-edge outcomes. The validation of this deep learning model encompasses an evaluation of its performance using the dataset. This process encompasses several stages, including data preprocessing, data partitioning, model training, performance assessment using a validation set, and testing the model's performance on an entirely distinct test set. The data was split into 80:20 ratio; 80 % for training and 20 % for testing. This systematic approach is integral in ensuring the model's ability to generalize to new data and produce dependable, accurate results. Proper data preprocessing is essential to enable effective learning by the model, which may encompass tasks like scaling, normalization, augmentation, and various data transformations. Subsequently, the collected data is bifurcated into two sets, namely the training set and the testing set. The envisioned model is then trained using the training set, and its performance is rigorously evaluated on the testing set. The procedural steps for validating a deep learning model using the dataset are depicted in Fig 5. After applying different transfer learning models (VGG16, Xception, EfficientNet, Inception, and ResNet50) the best acquired accuracy result was 98.09 % by ResNet50 which is shown in Fig 6.

## Limitations

None.

## Ethics Statement

This article does not involve any research involving human or animal subjects by any of the authors. The datasets utilized in this article are publicly accessible. When utilizing these datasets, it is essential to adhere to appropriate citation guidelines.

## CRediT authorship contribution statement

**Md Ripon Sheikh:** Investigation, Software, Validation, Formal analysis, Resources, Data curation, Writing – original draft, Visualization. **Md. Anwar Hossain:** Investigation, Software, Validation, Resources, Formal analysis, Data curation, Visualization, Writing – original draft. **Moazzem Hossain:** Investigation, Formal analysis, Resources, Data curation. **Md. Masudul Islam:** Project administration, Conceptualization, Methodology, Formal analysis, Resources, Writing – original draft, Visualization. **Galib Muhammad Shahriar Himel:** Project administration, Supervision, Conceptualization, Methodology, Formal analysis, Resources, Writing – original draft, Writing – review & editing.

## Data Availability

BananaVista: Banana Varieties Image Dataset (Original data) (Mendeley Data).
